# Influence of Media Composition on the Level of Bovine Satellite Cell Proliferation

**DOI:** 10.3390/ani13111855

**Published:** 2023-06-02

**Authors:** Karolina Zygmunt, Agnieszka Otwinowska-Mindur, Katarzyna Piórkowska, Wojciech Witarski

**Affiliations:** 1 Department of Animal Molecular Biology, National Research Institute of Animal Production, Krakowska 1, 32-083 Balice, Poland; katarzyna.piorkowska@iz.edu.pl; 2 Department of Genetics, Animal Breeding and Ethology, University of Agriculture in Krakow, Mickiewicza 24/28, 30-059 Krakow, Poland; agnieszka.otwinowska@urk.edu.pl

**Keywords:** proliferation, media composition, bovine satellite cells

## Abstract

**Simple Summary:**

Culturing of muscle precursor cells—satellite cells—is the basis for research on obtaining meat using in vitro techniques, which can become an alternative to traditional meat production. The available literature data do not give a clear answer as to the medium used at the proliferation stage of bovine satellite cells; therefore, there is a need to select the level of components of the growth medium. In our research, based on the fluorometric measurement of proliferating cell growth and qPCR analysis, we examined the effect of the three most common components: glucose, serum (bovine or horse), and bFGF—a mitogenic factor. The results show that the most critical component affecting the proliferation rate is the serum—the addition of bovine serum, followed by the addition of bFGF—at 10 ng/mL. In turn, the lower glucose content ensures the maintenance of cells at the early stage of myogenesis and, thus, the self-renewal of their population. In conclusion, a higher proliferation rate of bovine satellite cells is ensured under the conditions of bovine serum and a mitogenic factor at 10 ng/mL.

**Abstract:**

It is predicted that already in 2040, 35% of requirements for meat will be provided by in vitro production. Recreating the course of myogenesis in vitro, and thus resembling a structure of muscle tissue, is the basis for research focusing on obtaining cultured meat and requires providing relevant factors supporting the proliferation of satellite cells—being precursors of skeletal muscles. The present work aimed to develop the composition of the medium that would most effectively stimulate the proliferation of bovine satellite cells (BSCs). The modeling and optimization methods included the measurements of the synergistic, co-stimulatory effect of three medium components: the amount of glucose, the type of serum (bovine or horse), and the amount of mitogenic factor—bFGF. Additionally, the qPCR analyses determined the expression of genes involved in myogenesis, such as Pax7 and Myogenic Regulatory Factors, depending on the level of the tested factor. The results showed significant positive effects of serum type (bovine serum) and mitogenic factor (addition of 10 ng/mL bFGF) on the proliferation rate. In turn, qPCR analysis displayed no significant differences in the relative expression level of Pax7 genes and MRF factors for both factors. However, a statistically higher Pax7 and Myf5 gene expression level was revealed when a low glucose medium was used (*p* < 0.05). In conclusion, the components of the medium, such as bovine serum and the addition of a mitogenic factor at the level of 10 ng/mL, ensure a higher proliferation rate of BSCs and lower glucose content ensured the expression of crucial genes in the self-renewal of the satellite cell population.

## 1. Introduction

The world population is predicted to reach approximately 9.7 billion people in 2050 [1], so the demand for food—including meat—will continue to increase, contrary to the ability to produce the resources necessary for its production. Conventional meat production is associated with a destabilizing effect on the environment—highly impoverishing water resources and using vast areas—as much as 2/3 of available agricultural land—for pastures and meadows for grazing livestock [2]. Additionally, traditional meat farming leads to high greenhouse gas emissions—mainly carbon dioxide, nitrous oxide, and methane—which constitute 14% of total greenhouse gas emissions [3]. However, recent scientific reports show that cultured meat production using highly purified culture media can generate significantly higher carbon dioxide emissions than traditional meat husbandry. Solving the endotoxins problem or using cheaper substitutes for media components made from agricultural by-products would significantly reduce production costs [4]. In the context of a direct impact on human health, meat obtained by conventional methods poses risks of zoonotic pathogens and pesticides, fungicides or heavy metals used in agriculture for the production of fodder may be toxic. Therefore, in vitro meat production (named alternative “cultured meat”, “artificial meat”, or “lab-grown meat”) can be more environmentally friendly (by using less water and land), safer, and healthier for consumers alternative to traditional husbandry, due to much lower exposure to zoonotic pathogens such as *Salmonella*, *E. coli* or *Campylobacter* [5]. The in vitro technology also creates the possibility of enriching the composition of the product with nutrients that improve the quality and healthiness of cultured meat [6]. Using the ability of muscle stem cells to proliferate, differentiate, and self-renew their population, it is possible to construct three-dimensional structures in vitro that correspond to muscle tissue. It is predicted that already in 2040, 35% of requirements for meat will be provided by in vitro production [7].

As precursors of myofibers, muscle satellite cells (MuSCs) were first described by Alexander Mauro [8], who observed their presence between the basement membrane and the sarcolemma of myofibers dissected from the tibialis anticus muscle of an adult frog by electron microscopy. Satellite cells play a crucial role in the myogenesis process—both at the prenatal stage—when they are involved in muscle formation, and at the postnatal stage, in the regeneration of damaged skeletal muscles. During the organism’s homeostasis, MuSCs are in a quiescent state—in the G_0_ phase of the cell cycle, being mitotically inactive. Because of activation, in the form of mechanical damage to the muscle, they re-enter the G_1_ phase of the cell cycle. Gene *Pax7* is expressed in quiescent satellite cells; during MuSCs activation, additional genes such as genes from the family of myogenic regulatory factors (MRFs)—*Myf5* and *MyoD*—are beginning to be expressed. Activated satellite cells intensively proliferate to myoblast form and differentiate into myotubes, which is associated with *Myf6* and *MyoG* expression [9,10,11].

Recreating the course of myogenesis in vitro and thus resembling a structure of muscle tissue is the basis for research focusing on obtaining cultured meat. In the available literature, exist some combinations of medium components at the proliferation stage of the culture of bovine satellite cells (BSCs), and the inconsistency between their composition makes it difficult to select the optimal composition of the medium that supports the proliferation process (Table 1). So far, the conducted research has focused mainly on the selection of the medium at the stage of muscle cell differentiation [12,13]. Our research aimed to develop the optimal composition of the medium that would most effectively stimulate the proliferation of BSCs. In this case, achieving a high proliferation rate with a low degree of muscle progenitor cells that undergo spontaneous differentiation is important. The tested components were the glucose content, the type of serum and the concentration of the mitogenic factor—bFGF—which are mainly used to cultivate MuSCs. Culture media usually require serum supplementation and depend on cell type also other substances that provide cells with an additional reservoir of biologically active factors such as cytokines or hormones. The most popular type of serum is fetal bovine serum, but many studies on satellite cells also use horse serum [14,15,16]. Moreover, other components such as growth factors may stimulate dividing cells. bFGF—basic fibroblast growth factor—is a known mitogenic factor, inhibiting the process of satellite cell differentiation [17]. In the study, we used the modeling and optimization method—Design of experiment (DOE)—full fractional design, which allows for studying the influence of several factors on the response and interactions between the tested factors [18]. Additionally, an analysis of the expression of key genes involved in myogenesis—*Pax7* and Myogenic Regulator Factors—MRF factors: *Myf5*, *MyoD*, *Myf6* and *MyoG*, depending on the level of a particular component of the medium, was performed.

## 2. Materials and Methods

### 2.1. Isolation of Muscle Satellite Cells

Satellite cells were isolated from musculus longissimus dorsi from female calf specimen as two separate isolations (described as R1 and R2). A sample of skeletal muscles was sourced from fresh slaughter waste and therefore required no ethical committee acceptance according to local law. The tissue was digested with collagenase II (2 mg (500 U)/mL; 2–3 mL/g tissue; Thermo Fisher Scientific, Waltham, MA, USA) in Phosphate Buffered Saline—PBS buffer pH 7.2 (Thermo Fisher Scientific) with 4% BSA (Thermo Fisher Scientific) for two hours at 37 °C. Then, cells were washed in PBS buffer and filtered through a 70 µm nylon strainer (Corning, New York, NY, USA). Cells were centrifuged for 10 min at 500× *g*, the supernatant was discarded, and cells were purified by pre-plating method, based on the rate of adhesion of different types of cells to the surface. Cells were seeded in Skeletal Muscle Cell Medium (Cell Applications, Inc., San Diego, CA, USA) with 1% of antibiotic Primocin (InVivoGen, San Diego, CA, USA) on non-coated culture dish. After 1 h, the medium containing non-adherent cells were collected, centrifuged, and the cells were plated in the same medium on a thin layer of 0,1% gelatin (Thermo Fisher Scientific) [23]. The procedure was repeated during the next cell split. Part of the cells was frozen in a medium DMEM (Thermo Fisher Scientific) with the addition of 20% fetal bovine serum (Thermo Fisher Scientific), 1% antibiotic Primocin (InVivogen), and 10% DMSO (Thermo Fisher Scientific). 

### 2.2. Selection of the Components of the Tested Media

The experimental design was set to measure the synergistic, co-stimulatory effect of three medium components, which effect was evaluated on the proliferation rate of BSCs. Those stimulants were:

A—Glucose content in the culture medium—DMEM medium with low glucose content (1 g/L) versus DMEM medium with high glucose content (4.5 g/L) (Thermo Fisher Scientific).

B—Type of serum—horse serum (hs) at a concentration of 20% versus fetal bovine serum (fbs) at a concentration of 20% (Thermo Fisher Scientific). It was hypothesized that fetal bovine serum would stimulate the proliferation of BSCs more efficiently [24,25]. 

C—Content of bFGF (Thermo Fisher Scientific) with known mitogenic effect at concentrations of 5 ng/mL and 10 ng/mL. 

Culture media was prepared fresh just before each medium change due to the instability of bFGF.

During the entire experiment, sera from one lot were used due to the possibility of differences in the concentration of individual components between batches.

### 2.3. Cell Culture and Estimation of Proliferation Rate and Visualisation

Satellite cell lines R1 and R2 (both—3rd passage since isolation) were plated in the 96-well multiwell plate at a density of 3500 cells/wells in Skeletal Muscle Cell Medium (Cell Applications, Inc.) supplemented with 1% addition of antibiotic Primocin (InVivoGen) on a thin layer of 0.1% gelatin used as a coating (Thermo Fisher Scientific). Each cell line consisted of four replicates per experimental group. After 24 h, the number of cells in each well was estimated based on a fluorimetric method—CellTiter Blue Assay (Promega, Madison, WI, USA). The CellTiter solution was prepared according to the manufacturer’s protocol, then was added to the plate with treated cells and incubated for one hour in 5% CO_2_ in humidified air at 37 °C (previously length of the incubation period and the number of cells/wells were optimized). Fluorescence intensity was measured at 535 nm excitation and 595 nm emission wavelength using PlateReader AF2200 (Eppendorf, Hamburg, Germany). After the measurement, in each group tested media were added containing an appropriate amount of tested factors. The number of proliferating cells was estimated, based on CellTiter Blue assay, after three, five, seven, and ten days of incubation in the tested medium. The measurement results were obtained in the RFU unit—relative fluorescence unit. RFU values from each measurement were normalized based on day 0 RFU values. 

Alongside to described, microscopic observation of satellite cells was carried out. Pictures of the representative area were visualized in Opta-tech microscope using OptaView-IS programme. Further post-processing consisted of contrast enhancement for every picture using ImageJ CLAHE algorithm (blocksize 79, hist bins 50, max slope 2.5) [26,27]. 

### 2.4. Statistical Analysis

The results were analyzed using Python [28] with NumPy [29], SciPy [30], Statsmodels [31], Pandas [32,33], and Matplotlib [34]. A 2^3^ full factorial design was applied to evaluate the effects of three factors (Xi) on experimental results. The high and low levels defined for the 2^3^ factorial designs are presented in Table 2. The variables X_1_, X_2,_ and X_3_ represented the levels of glucose, serum, and bFGF, respectively. The analysis was performed separately for R1 and R2 cell lines and on the subsequent day of the experiment (3, 5, 7, or 10), so eight designs of experiments (DoE) were created. Additionally, the design is replicated fourth time. The experimental conditions obtained for each reaction set by the full factorial design are shown in Table 3. Every treatment factor in an experiment has two levels. The higher level (maximum) was designated as (+1) and the lower value (minimum) was designated as (−1). 

The coded mathematical model may be given as:

Y = α_0_ + α_1_X_1_ + α_2_X_2_ + α_3_X_3_ + α_12_X_1_X_2_ + α_13_X_1_X_3_ + α_23_X_2_X_3_ + α_123_X_1_X_2_X_3_

where Y is the proliferation rate measured as RFU value, X_i_ are the coded variables (−1 or +1), and α_i_ represents the fitted constants. Additionally, in this notation the α_ij_ constant represented the estimation of the interaction effect between factor i and j for the response.

The *p*-values are used as a tool to check the significance of each coefficient, which can indicate the pattern of the interactions between the independent variables. If the *p*-value is below 0.05, then the model is significant at the 95% confidence interval. Additionally, the Akaike Information Criterion (AIC), Bayesian Information Criterion (BIC), coefficient of determination (R^2^), and adjusted coefficient of determination (R^2^_adj_) were calculated to show the goodness of fit of models.

Pareto’s charts were used to evaluate the meaning and type of effects. Pareto analysis indicates the relative importance of each independent model’s parameter and their interactions on the proliferation rate measured as RFU value. The values displayed in the horizontal columns are the absolute values of t-Student test values for each effect and the vertical line indicates the minimal significant effect magnitude for a 95% confidence level.

At the end of the analysis, the statistical validation of the model was performed using an analysis of variance (Table 4). The main effect is the mean of all of the responses produced by changing the level of a factor, i.e., glucose, serum, or bFGF.

### 2.5. Analysis of the Expression of Genes of Myogenesis

For the analysis of myogenesis gene expression, the effect of each component was examined individually. Satellite cells (3rd passage) were plated on 35 mm culture dishes at a density of 70,000 cells/dish in Skeletal Muscle Cell Medium (Cell Applications, Inc.) supplemented with 1% of antibiotic Primocin (InVivoGen) on a thin layer of 0.1% gelatin (Thermo Fisher Scientific), six replicates per group, within the tested factor. After 24 h, the medium was replaced with the tested medium depending on the group studied. After three days, three replicates from each group were harvested, and RNA was isolated according to the manufacturer’s protocol using PureLink RNA Mini Kit (Thermo Fisher Scientific). The medium was changed to a new one in the residual culture plates and after five days, RNA was isolated as described above. RNA concentration was determined using a NanoDrop2000C (Thermo Fisher Scientific) spectrophotometer, and the integrity of the isolated RNA was checked electrophoretically on a 1.5% agarose gel. Reverse transcription was performed using a High Capacity cDNA Reverse Transcription Kit (Thermo Fisher Scientific). Before starting the analysis, all tested solutions were equilibrated, regarding RNA concentrations in RNase-free water. The qPCR method was used to determine the expression of *Pax7*, *Myf5*, *MyoD*, *Myf6*, and *MyoG* genes. The *RPL27* and *OAZ1* genes were the endogenous control. The primer sequences were designed in Primer3Web (v. 4.1.0, https://primer3.ut.ee/, accessed on 30 November 2022) and produced by Genomed (Warsaw, Poland) (Table 5). Gene expression analysis was performed using the HS-PCR Mix SYBR according to the manufacturer’s protocol (A&ABiotechnology, Gdansk, Poland) and QuantStudio 7 Pro System (Thermo Scientific).

### 2.6. Statistical Analysis of the Expression of Genes 

Statistical analysis of the obtained results was carried out in the Statistica program (StatSoft, v.13). The normal distribution was tested with the Shapiro–Wilk test. The analyzes were based on the one-way analysis of variance (ANOVA) using the Bonferroni post hoc test. In the case of failure to comply with assumptions of Leavene’s test, the Mann–Whitney U test was used.

## 3. Results

### 3.1. Full Factorial Design of Tested Components 

**Figure 1:**
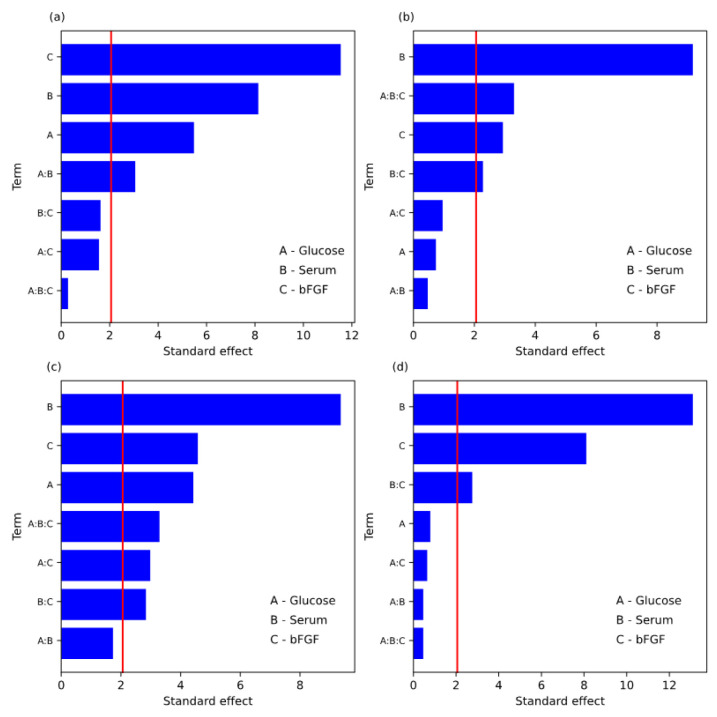
Pareto chart for standard effect; cell line R1; (**a**)-3rd day, (**b**)-5th day, (**c**)-7th day, (**d**)-10th day. Red vertical line correspondences to a significance level of α = 0.05.

On the third day, in cell line R1 (Table 6 and Figure 1a), the concentration of bFGF in the medium (component C) showed the most robust effect strength of 6.92 × 10^3^ value from among tested components. All the effects of the main tested, i.e., glucose (A), serum (B), and bFGF (C) components, were significant (*p* < 0.05). The interaction of the two A:B factors was significant (*p* < 0.05). The obtained results indicate that component C is a key factor in stimulating the proliferation of satellite cells and that its level is independent of the levels of other tested components on the third day. According to the data presented in Table 6 and the graphical Pareto chart shown in Figure 1a, the final first-order model for RFU in terms of coded parameters can be proposed. What is important, the nonsignificant effects were neglected to form the best-fitted regression equation, so the proposed model was:

RFU value at 3rd day = 22,514.38 + 1642.69X_1_ + 2438.38X_2_ + 3458.25X_3_ + 915.44X_1_X_2_

On days 5, 7, and 10, in cell line R1 (Table 7, Table 8 and Table 9 and Figure 1b–d), the serum type (component B) showed the most robust effect strength of 6290.25; 4874.313; 6903.375 values from among tested components, respectively. The type of serum was the most important component affecting the proliferation of BSCs. The factors that occur with a *p*-value less than 0.05 are considered to have a significant effect on the response significantly. On days 5 and 10, the main effects of components B and C were significant (*p* < 0.05), and on day 7, all the tested components, i.e., glucose (A), serum (B), and bFGF (C) were significant (*p* < 0.05). On days 5, 7, and 10, the interaction of factors B:C was significant, and on day 7, the interaction of factors A:C was significant. On days five and seven, the interaction of all three factors A:B:C was significant. Thus, according to the data presented in Table 7, Table 8 and Table 9 and in Figure 1b–d, the regressions model for RFU in terms of coded parameters were:

RFU value at the 5th day = 34,584.50 + 3145.13X_2_ − 1007.56X_3_ + 783.44X_2_X_3_ + 1132.19X_1_X_2_X_3_,

RFU value at the 7th day = 31,204.28 − 1151.03X_1_ + 2437.16X_2_ + 1191.28X_3_ + 776.34X_1_X_3_ + 738.16 X_2_X_3_ + 857.09X_1_X_2_X_3_,

RFU value at the 10th day = 38,333.69 + 3451.69X_2_ + 2134.81X_3_ + 727.44X_1_X_2_.

Four of the goodness of fit parameters, i.e., AIC, BIC, R^2^ and R^2^_adj_ were calculated. The model with the lowest AIC and BIC and with higher, closer to 1 R^2^ and R^2^_adj_ had better fit to data. In the case of R1 cell line, the obtained statistical parameters are presented in Table 6, Table 7, Table 8 and Table 9 and show a better fit on the 3rd and 10th days.

**Figure 2:**
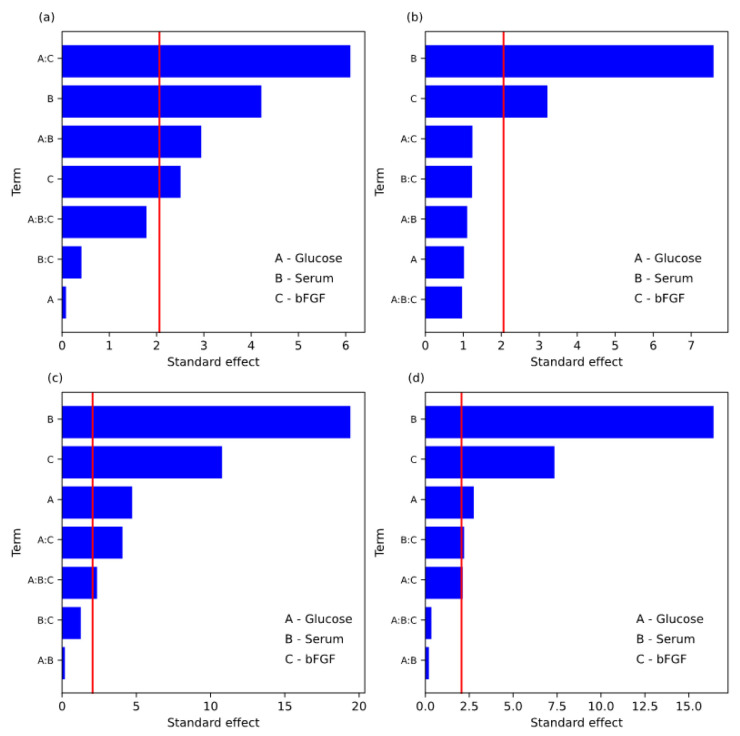
Pareto chart for standard effect; cell line R2; (**a**)-3rd day, (**b**)-5th day, (**c**)-7th day, (**d**)-10th day. Red vertical line correspondences to a significance level of α = 0.05.

On days 3, 5, 7, and 10, in cell line R2 (Table 10, Table 11, Table 12 and Table 13 and Figure 2), the serum type (component B) showed the most robust effect strength of values of 1221.5, 4393.375, 6027.438, and 6903.375 among tested components, respectively. On days 3, 5, and 10, the main effects of components B and C were significant, and on day 7, the main effects of all components (i.e., A, B, C) were significant. On day three, the two interactions between factors A:B and A:C were significant, whereas on days 7 and 10, the interaction between factors A:C and B:C was significant. Moreover, on day seven, the interaction of the three factors A:B:C was also significant. The proposed equations of the final first-order models for RFU in term of coded parameters on each day were:

RFU value at 3rd day = 9487.44 + 610.75X_2_ − 363.13X_3_ − 426.38X_1_X_2_ − 883.63X_1_X_3_,

RFU value at the 5th day = 17,818.31 + 2196.69X_2_ + 930.69X_3_,

RFU value at the 7th day = 20,728.22 − 733.09X_1_ + 3013.72X_2_ + 1672.72X_3_ − 632.09X_1_X_3_ − 730.1875X_1_X_2_X_3_,

RFU value at the 10th day = 27,029.34 − 846.28X_1_ + 5033.34X_2_ + 2254.59X_3_ − 651.53X_1_X_3_ + 677.34X_2_X_3_.

The goodness of fit parameters, i.e., AIC, BIC, R^2^, and R^2^_adj_ of R2 cell line, are presented in Table 10, Table 11, Table 12 and Table 13 and shows better fit on the 7th and 10th days.

**Figure 3:**
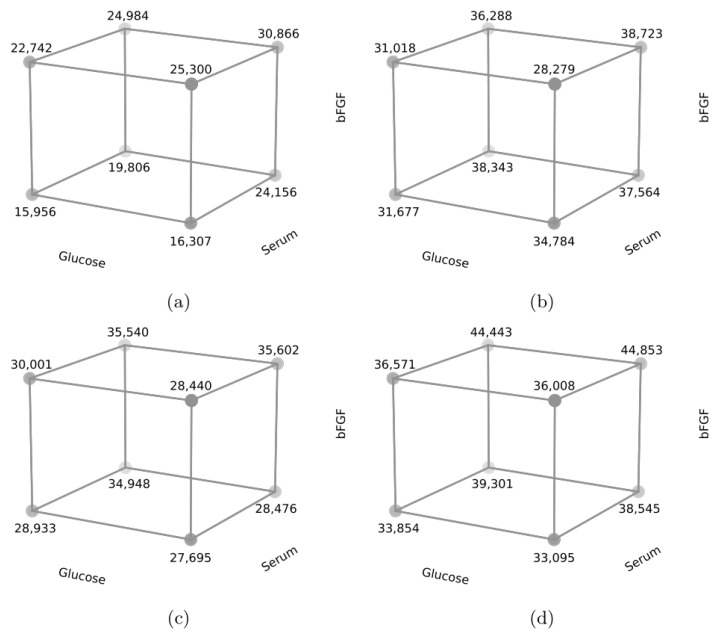
Cube plot for media components, cell line R1; (**a**)-3rd day, (**b**)-5th day, (**c**)-7th day, (**d**)-10th day.

**Figure 4:**
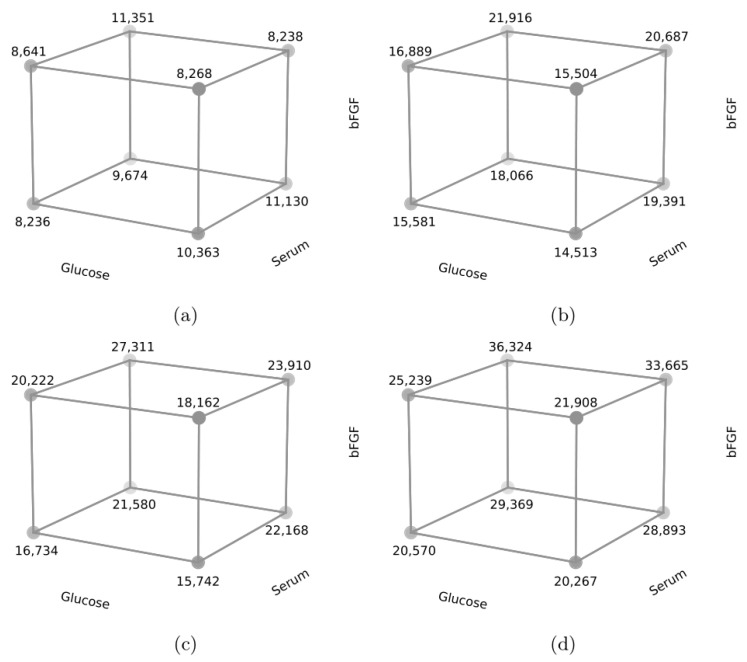
Cube plot for media components, cell line R2; (**a**)-3rd day, (**b**)-5th day, (**c**)-7th day, (**d**)-10th day.

In addition, for an evaluation of models and the relationship between factors A, B, C and response and cube plots, as shown in Figure 3 and Figure 4, were generated. In cell line R1 (Figure 3), the highest RFU value was obtained for bovine serum and 10 ng/mL bFGF and on the third and seventh days also for high glucose content. On day three, higher RFU values were obtained primarily for the higher level of component C—10 ng/mL bFGF (*p* < 0.05). The level of component B—the type of medium, where bovine serum gave higher values (*p* < 0.05), and the level of component A—glucose level—where high glucose content resulted in higher values also had a significant effect (*p* < 0.05). On days 5, 7, and 10, higher RFU values were observed primarily in the group with a higher value of component B—bovine serum (*p* < 0.05). An important component was also bFGF (component C), where using a higher dose—10 ng/mL—resulted in higher RFU values (*p* < 0.05). On day seven, a significant effect was also shown by the glucose level in the medium—component A, where higher glucose content resulted in higher RFU values (*p* < 0.05).

In cell line R2 (Figure 4), the highest RFU value was obtained for bovine serum (component B) and 10 ng/mL bFGF (component C), and on days 7 and 10 also for low glucose content (component A). Higher RFU values were obtained mainly for component B—bovine serum (*p* < 0.05), but the level of component C also had a significant effect—bFGF—at the level of 10 ng/mL (*p* < 0.05). On days 7 and 10, component A—glucose content also had a significant effect, where lower levels resulted in higher RFU values (*p* < 0.05).

### 3.2. Microscopic Observations of Proliferating BSCs

**Figure 5:**
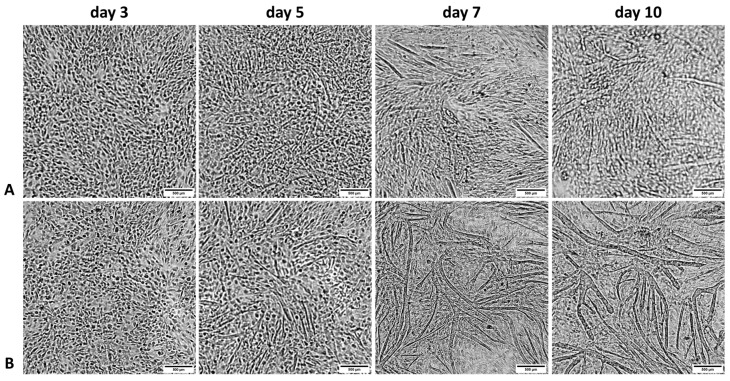
Microscopic observations of proliferating satellite cells (representative selection) (**A**)—satellite cells cultured in a medium with high glucose content, 20% addition of bovine serum, and 10 ng/mL bFGF; (**B**)—cell culture of satellite cells in a medium with low glucose content and 20% addition of horse serum. Scalebar represents 500 µm.

Microscopic observation showed that in the group treated with bovine serum and 10 ng/mL bFGF addition, cell differentiation was lower than in the group with horse serum and 5 ng/mL bFGF addition (Figure 5). There formation of myotubes was observed already on the fifth day of the experiment.

### 3.3. Gene Expression: Genes Involved in Myogenesis

**Figure 6:**
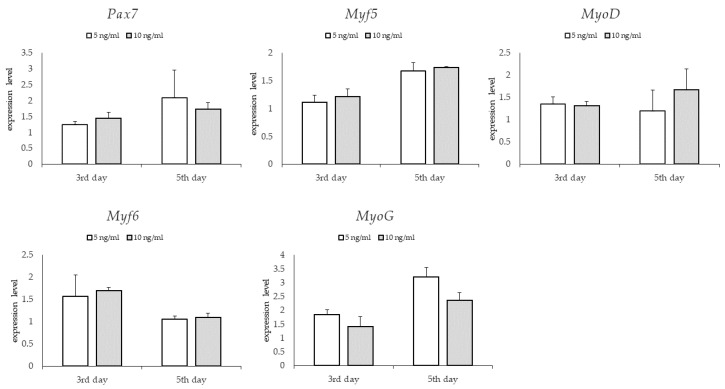
Quantitative PCR analysis of specific markers for muscle satellite cells maintenance in tested media differing in the amount of bFGF.

**Figure 7:**
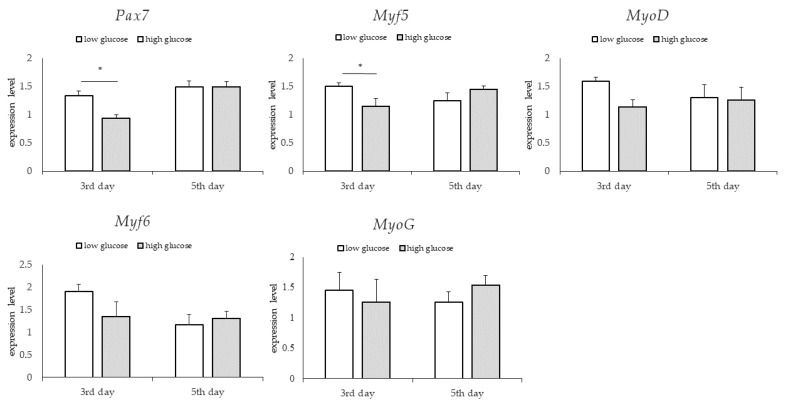
Quantitative PCR analysis of specific markers for muscle satellite cells maintenance in tested media differing in the amount of glucose; *p* < 0.05 *.

**Figure 8:**
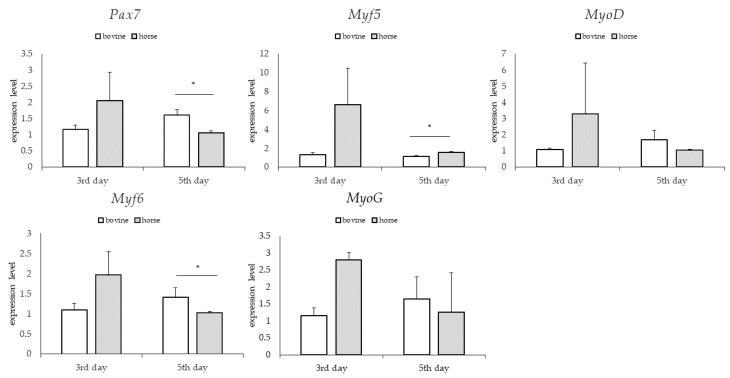
Quantitative PCR analysis of specific markers for muscle satellite cells maintenance in tested media differing in the type of serum; *p* < 0.05 *.

For the groups differing in bFGF content, no significant differences were found in the relative expression of the examined genes on the third and fifth days (Figure 6). For groups differing in glucose content, significantly higher levels of relative expression of *Pax7* and *Myf5* genes—genes expressed at the early stage of myogenesis—were found in the group supplemented with lower glucose content on the third day (*p* < 0.05). On the fifth day, no significant differences in the relative expression of the tested genes were observed between the study groups (Figure 7). For the groups differing in the type of serum, no significant differences in the relative expression of the examined genes were found on the third day. However, large standard deviations were observed in the group treated with horse serum. On the fifth day, a significantly higher relative level of *Pax7* and *Myf6* gene expression was observed in the bovine serum-treated group and a significantly higher relative expression level of *Myf5* gene expression in the horse serum-treated group (*p* < 0.05) (Figure 8).

## 4. Discussion

Recreating the process of myogenesis in vitro requires the provision of relevant factors supporting the proliferation of satellite cells, which are contained in the culture medium. Our research aimed to develop the composition of the culture medium that would most efficiently support the proliferation of BSCs. The experimental design was set to measure the synergistic, co-stimulatory effect of three medium components: glucose content, type of serum (bovine or horse), and mitogenic factor—bFGF—content. Each observation (except observation for 3rd day, cell line R1) showed that the type of serum had the most robust effect on the proliferation rate of BSCs amongst tested components. The main effect of the tested component was statistically significant (*p* < 0.05), and in addition, in the majority of the observations, the interaction between component B (serum type) and component C (bFGF) was statistically significant (*p* < 0.05). In the present study, bovine serum was proven to stimulate the proliferation of BSCs to a greater extent than horse serum. Our observations are consistent with the study by Woods et al. [35], which showed a higher proliferation rate of BSCs in a medium supplemented with bovine serum than horse serum 24 h after cell seeding. Research by Franke et al. [25] focusing on comparing the effect of bovine and horse serum on the proliferation and differentiation of equine bronchial fibroblasts showed that bovine serum more efficiently supported the proliferation process of fibroblasts by maintaining normal cell morphology and increasing the number of passages. Similar observations were made in the case of equine mesenchymal stromal cells, where cells cultured in bovine serum proliferated faster and secreted much more, e.g., bFGF and interleukins (IL-4, IL-5, IL-17) [24]. Gene expression analysis did not provide conclusive information on which of the sera maintained the expression profile characteristic of the early stage of myogenesis. A statistically higher expression level of *Pax7* and *Myf6* genes was observed in the group treated with bovine serum (*p* < 0.05). *Pax7* and *Myf6* genes expression occur at different stages of myogenesis, in the early and late stages, respectively. Quiescent satellite cells express *Pax7*, which maintains cells in an undifferentiated state and regulates genes involved in the initiation of proliferation and inhibiting differentiation [8,36]. The expression of *Myf6* gene—supervises the inhibition of premature differentiation and is maintained at a state level [37,38]. However, it is worth noting that the standard deviations within the group treated with horse serum were high, which may suggest that bovine serum provides more stable conditions for cell proliferation. 

Basic fibroblast growth factor (bFGF) can regulate proliferation and myogenesis by stimulating or inhibiting muscle metabolic pathways [39]. Studies conducted on Jeju Black pig muscle cells showed that the presence of bFGF affected the rate of proliferation and, together with EGF, also the level of expression of the *MyoD* gene [40]. The present study confirmed the pro-mitogenic effect of bFGF on BSCs and after the serum type showed the most robust effect on the proliferation rate. Each observation showed that the factor’s main influence was statistically significant (*p* < 0.05). Interactions between component C (bFGF) and component B (serum type) in most observations were statistically significant. In the R2 cell line, a statistically significant interaction between component A (glucose) and component C (bFGF) content was observed on the 3rd, 7th and 10th days (*p* < 0.05). In greater observations, the addition of 10 ng/mL bFGF resulted in a significantly higher rate of proliferation than the addition of 5 ng/mL. Higher concentrations of bFGF were not tested due to the need to select the two most commonly used levels by reason of the mathematical model used. Moreover, microscopic observations showed that in the group treated with bovine serum and 10 ng/mL bFGF differentiation was lower than in the group with horse serum and 5 ng/mL bFGF, which is consistent with the rest of the observations. This confirms the fact that using bovine serum together with bFGF at the level of 10 ng/mL more effectively supports the proliferation process.

Recent research shows that muscle cells’ proliferative and self-renewal capacities are greater in a medium with a lower glucose content [41]. Moreover, high glucose concentrations may induce adipogenic differentiation in satellite cells [42]. This examination could be successfully used in a selection of the composition of the medium for co-cultures of satellite cells with adipocytes in seeking to obtain cultured meat. In this study, generally, we observed no significant effect associated with glucose content on the proliferation rate in BSCc compared to the other components. Notably, the expression of *Pax7* and *Myf5* genes—genes expressed at the early stages of myogenesis—was significantly higher in the group supplemented with lower glucose content on the third day. The observation is consistent with the research of Furuichi et al. [41] and proves the ability of satellite cells to self-renew in conditions of lower glucose content. These results would suggest using a medium with low glucose content, which provides higher expression levels of genes involved in the self-renewal of satellite cell populations. 

It is worth noting that cell cultivation based on serum of animal origin is contrary to the ideology of cultured meat, raises many ethical controversies, and requires high financial investments. Research is still undertaken to find optimal substitutes for animal serum because serum-free cultures are still inefficient [43]. In 2022, Stout et al. [44] proposed the Beefy-9—B8 medium supplemented with recombinant albumin—which allowed the cultivation of BSCs for seven passages. Independently, in 2022, Kolkmann et al. [19] developed a serum-free medium (SFM) composition supporting proliferation almost as efficiently as the medium supplemented with animal serum. However, both solutions still require a lot of studies, although they optimistically approach us closer to serum-free cell cultivation, which is free of suffering for animals.

## 5. Conclusions

The component which most significantly affects the level of BSCs proliferation is the type of serum. Using bovine serum provides a higher rate of proliferation and based on qPCR analysis, it would seem that there are more stable growth conditions for cells than horse serum. The second significant component is bFGF, where adding 10 ng/mL significantly increased the proliferation rate. In most observations, glucose does not significantly affect the rate of proliferation. However, the low glucose content ensured the expression of crucial genes in the self-renewal of the satellite cell population, which is consistent with previous studies. The highest rate of proliferation measured as RFU value was observed, supposing the addition of bovine serum and bFGF at the concentration of 10 ng/mL.

## Figures and Tables

**Table 1:** Summary of compositions of media for BSCs cultivation.

Composition of Media for BSCs Cultivation	References
Ham’s F-10 Nutrient Mix or DMEM/F-12 as basal medium, supplemented with 20% fbs and 5 ng/mL FGF-2	[19]
Low-glucose DMEM as basal medium, supplemented with 10% hs	[20]
DMEM/F12 as basal medium, supplemented with 0.02 M glutamine and 10% fbs	[12,21]
DMEM as a basal medium, supplemented with 20% fbs, and 5 ng/mL basic FGF	[22]

**Table 2:** Factors and levels used in 2^3^ factorial designs.

Variables	Factors	Minimum Value (−1)	Maximum Value (+1)
X_1_	Glucose	Low	High
X_2_	Serum	20% hs	20% fbs
X_3_	bFGF	5 ng/mL	10 ng/mL

fbs—fetal bovine serum, hs—horse serum.

**Table 3:** Experimental design matrix and mean experiment results.

	Glucose		Serum		bFGF		Day			
Experiment	(A)	X_1_	(B)	X_2_	(C)	X_3_	3	5	7	10
Cell R1										
1	Low	−1	20% hs	−1	5	−1	15,955.75	31,677.25	28,933.00	33,853.67
2	High	1	20% hs	−1	5	−1	16,306.75	34,783.50	27,695.00	33,094.92
3	Low	−1	20% fbs	1	5	−1	19,805.75	38,343.00	34,947.75	39,300.67
4	High	1	20% fbs	1	5	−1	24,156.25	37,564.50	28,476.25	38,544.92
5	Low	−1	20% hs	−1	10	1	22,741.75	31,017.50	30,000.75	36,570.17
6	High	1	20% hs	−1	10	1	25,299.75	28,279.25	28,439.75	36,007.92
7	Low	−1	20% fbs	1	10	1	24,983.50	36,288.25	35,539.75	44,442.42
8	High	1	20% fbs	1	10	1	30,865.50	38,722.75	35,602.00	44,852.17
Cell R2										
1	Low	−1	20% hs	−1	5	−1	8235.83	15,581.58	16,733.53	20,570.58
2	High	1	20% hs	−1	5	−1	10,363.08	14,512.83	15,741.53	20,267.58
3	Low	−1	20% fbs	1	5	−1	9674.08	18,065.83	21,579.53	29,369.08
4	High	1	20% fbs	1	5	−1	11,130.58	19,391.58	22,167.53	28,893.08
5	Low	−1	20% hs	−1	10	1	8640.83	16,889.08	20,221.50	25,239.33
6	High	1	20% hs	−1	10	1	8268.33	15,504.33	18,161.50	21,907.83
7	Low	−1	20% fbs	1	10	1	11,351.08	21,916.33	27,310.75	36,324.83
8	High	1	20% fbs	1	10	1	8238.33	20,687.58	23,910.00	33,665.08

fbs—fetal bovine serum; hs—horse serum.

**Table 4:** Analysis of variance (*p*-values) for cell lines R1 and R2 in subsequent day of experiment.

Source	Cell Line R1				Cell Line R2			
	Day 3	Day 5	Day 7	Day 10	Day 3	Day 5	Day 7	Day 10
Main effects	2.00 × 10^−12^	1.94 × 10^−8^	8.79 × 10^−10^	1.35 × 10^−12^	7.12 × 10^−4^	3.07 × 10^−7^	2.39 × 10^−16^	3.56 × 10^−14^
2-way interactions	9.29 × 10^−3^	1.24 × 10^−1^	1.99 × 10^−3^	6.47 × 10^−2^	8.76 × 10^−6^	5.17 × 10^−2^	3.17 × 10^−3^	4.37 × 10^−2^
3-way interaction	7.81 × 10^−1^	2.99 × 10^−3^	3.06 × 10^−3^	6.50 × 10^−1^	8.69 × 10^−2^	6.58 × 10^−4^	2.72 × 10^−2^	7.34 × 10^−1^

If the *p* < 0.05, then the source is significant.

**Table 5:** Primer sequences for studied genes.

Gene	Starter Forward	Starter Reverse	Length of the Product
*Pax7*	5′ AAGCGGACAAGAAGGAGGAG 3′	5′ CGGGTTCTGACTCCACATCT 3′	114
*Myf5*	5′ TGCTTAGGGAACAGGTGGAA 3′	5′ AACTGCTGCTCTTTCTGGAC 3′	135
*MyoD*	5′ AACACTACAGCGGCGACT 3′	5′ GTAGTAAGTGCGGTCGTAGC 3′	122
*Myf6*	5′ CCCTTCAGCTACAGACCCAA 3′	5′ CCTTGGCAGTTATCACGAGC 3′	118
*MyoG*	5′ TCCAGTACATAGAGCGCCTG 3′	5′ CTATGGGAGCTGCATTCACTG 3′	121
*RPL27*	5′ ATAATCACCTCATGCCCACAA 3′	5′ CATGACCTTTGCCTCTCGTC 3′	206
*OAZ1*	5′ TTCGCCAGAGAGAAGGAAGG 3′	5′ GGACCCAGGTTACTACAGCA 3′	143

**Table 6:** Estimated effects and coefficients for media components, day 3, cell line R1.

Term	Effect	Coefficient	SE Coef	t-Value	*p*-Value
Constant	4.50 × 10^4^	22,514.38	299.62	75.14	5.34 × 10^−30^
A	3.29 × 10^3^	1642.69	299.62	5.48	1.23 × 10^−5^
B	4.88 × 10^3^	2438.38	299.62	8.14	2.33 × 10^−8^
C	6.92 × 10^3^	3458.25	299.62	11.54	2.79 × 10^−11^
A:B	1.83 × 10^3^	915.44	299.62	3.06	5.44 × 10^−3^
A:C	9.35 × 10^2^	467.31	299.62	1.56	1.32 × 10^−1^
B:C	−9.73 × 10^2^	−486.50	299.62	−1.62	1.18 × 10^−1^
A:B:C	−168.875	−84.44	299.62	−0.28	7.81 × 10^−1^
AIC	BIC	R^2^	R^2^_adj_		
573.47	585.20	0.91	0.88		

**Table 7:** Estimated effects and coefficients for media components, day 5, cell line R1.

Term	Effect	Coefficient	SE Coef	t-Value	*p*-Value
Constant	6.92 × 10^4^	34,584.50	342.80	100.89	4.63 × 10^−33^
A	5.06 × 10^2^	253.00	342.80	0.74	4.68 × 10^−1^
B	6.29 × 10^3^	3145.13	342.80	9.17	2.57 × 10^−9^
C	−2.02 × 10^3^	−1007.56	342.80	−2.94	7.17 × 10^−3^
A:B	3.22 × 10^2^	161.00	342.80	0.47	6.43 × 10^−1^
A:C	−6.58 × 10^2^	−328.94	342.80	−0.96	3.47 × 10^−1^
B:C	1.57 × 10^3^	783.44	342.80	2.29	3.14 × 10^−2^
A:B:C	2264.375	1132.19	342.80	3.30	2.99 × 10^−3^
AIC	BIC	R^2^	R^2^_adj_		
582.09	593.81	0.82	0.77		

**Table 8:** Estimated effects and coefficients for media components, day 7, cell line R1.

Term	Effect	Coefficient	SE Coef	t-Value	*p*-Value
Constant	6.24 × 10^4^	31,204.28	260.24	119.90	7.40 × 10^−35^
A	−2.30 × 10^3^	−1151.03	260.24	−4.42	1.80 × 10^−4^
B	4.87 × 10^3^	2437.16	260.24	9.36	1.74 × 10^−9^
C	2.38 × 10^3^	1191.28	260.24	4.58	1.22 × 10^−4^
A:B	−9.03 × 10^2^	−451.28	260.24	−1.73	9.57 × 10^−2^
A:C	1.55 × 10^3^	776.34	260.24	2.98	6.46 × 10^−3^
B:C	1.48 × 10^3^	738.16	260.24	2.84	9.12 × 10^−3^
A:B:C	1714.188	857.09	260.24	3.29	3.06 × 10^−3^
AIC	BIC	R^2^	R^2^_adj_		
564.45	576.18	0.87	0.83		

**Table 9:** Estimated effects and coefficients for media components, day 10, cell line R1.

Term	Effect	Coefficient	SE Coef	t-Value	*p*-Value
Constant	7.67 × 10^4^	38,333.69	263.46	145.50	7.17 × 10^−37^
A	−4.17 × 10^2^	−208.38	263.46	−0.79	4.37 × 10^−1^
B	6.90 × 10^3^	3451.69	263.46	13.10	1.99 × 10^−12^
C	4.27 × 10^3^	2134.81	263.46	8.10	2.51 × 10^−8^
A:B	2.44 × 10^2^	121.88	263.46	0.46	6.48 × 10^−1^
A:C	3.41 × 10^2^	170.25	263.46	0.65	5.24 × 10^−1^
B:C	1.45 × 10^3^	727.44	263.46	2.76	1.09 × 10^−2^
A:B:C	242.25	121.13	263.46	0.46	6.50 × 10^−1^
AIC	BIC	R^2^	R^2^_adj_		
565.24	576.97	0.91	0.89		

**Table 10:** Estimated effects and coefficients for media components, day 3, cell line R2.

Term	Effect	Coefficient	SE Coef	t-Value	*p*-Value
Constant	1.90 × 10^4^	9487.44	144.90	65.48	1.43 × 10^−28^
A	2.46 × 10^1^	12.31	144.90	0.08	9.33 × 10^−1^
B	1.22 × 10^3^	610.75	144.90	4.22	3.06 × 10^−4^
C	−7.26 × 10^2^	−363.13	144.90	−2.51	1.94 × 10^−2^
A:B	−8.53 × 10^2^	−426.38	144.90	−2.94	7.11 × 10^−3^
A:C	−1.77 × 10^3^	−883.63	144.90	−6.10	2.68 × 10^−6^
B:C	1.19 × 10^2^	59.31	144.90	0.41	6.86 × 10^−1^
A:B:C	−517.375	−258.69	144.90	−1.79	8.69 × 10^−2^
AIC	BIC	R^2^	R^2^_adj_		
526.98	538.70	0.75	0.68		

**Table 11:** Estimated effects and coefficients for media components, day 5, cell line R2.

Term	Effect	Coefficient	SE Coef	t-Value	*p*-Value
Constant	3.56 × 10^4^	17,818.31	289.42	61.57	6.22 × 10^−28^
A	−5.89 × 10^2^	−294.56	289.42	−1.02	3.19 × 10^−1^
B	4.39 × 10^3^	2196.69	289.42	7.59	7.91 × 10^−8^
C	1.86 × 10^3^	930.69	289.42	3.22	3.70 × 10^−3^
A:B	6.38 × 10^2^	318.81	289.42	1.10	2.82 × 10^−1^
A:C	−7.18 × 10^2^	−358.81	289.42	−1.24	2.27 × 10^−1^
B:C	7.12 × 10^2^	355.94	289.42	1.23	2.31 × 10^−1^
A:B:C	−559.625	−279.81	289.42	−0.97	3.43 × 10^−1^
AIC	BIC	R^2^	R^2^_adj_		
571.25	582.98	0.76	0.68		

**Table 12:** Estimated effects and coefficients for media components, day 7, cell line R2.

Term	Effect	Coefficient	SE Coef	t-Value	*p*-Value
Constant	4.15 × 10^4^	20,728.22	155.25	133.51	5.63 × 10^−36^
A	−1.47 × 10^3^	−733.09	155.25	−4.72	8.42 × 10^−5^
B	6.03 × 10^3^	3013.72	155.25	19.41	3.52 × 10^−16^
C	3.35 × 10^3^	1672.72	155.25	10.77	1.12 × 10^−10^
A:B	5.98 × 10^1^	29.91	155.25	0.19	8.49 × 10^−1^
A:C	−1.26 × 10^3^	−632.09	155.25	−4.07	4.40 × 10^−4^
B:C	3.91 × 10^2^	195.72	155.25	1.26	2.20 × 10^−1^
A:B:C	−730.1875	−365.09	155.25	−2.35	2.72 × 10^−2^
AIC	BIC	R^2^	R^2^_adj_		
531.39	543.12	0.96	0.94		

**Table 13:** Estimated effects and coefficients for media components, day 10, cell line R2.

Term	Effect	Coefficient	SE Coef	t-Value	*p*-Value
Constant	5.41 × 10^4^	27,029.34	306.69	88.13	1.18 × 10^−31^
A	−1.69 × 10^3^	−846.28	306.69	−2.76	1.09 × 10^−2^
B	1.01 × 10^4^	5033.34	306.69	16.41	1.51 × 10^−14^
C	4.51 × 10^3^	2254.59	306.69	7.35	1.36 × 10^−7^
A:B	1.25 × 10^2^	62.34	306.69	0.20	8.41 × 10^−1^
A:C	−1.30 × 10^3^	−651.53	306.69	−2.12	4.41 × 10^−2^
B:C	1.35 × 10^3^	677.34	306.69	2.21	3.70 × 10^−2^
A:B:C	211.1875	105.59	306.69	0.34	7.34 × 10^−1^
AIC	BIC	R^2^	R^2^_adj_		
574.96	586.69	0.93	0.91		

## Data Availability

The data supporting reported results are in the possession of the authors (K.Z. and W.W.).

## References

[1] United Nations World Population Prospects: The 2019 Revision. Population Database. https://population.un.org/wpp/.

[2] FAO-FAOSTAT (2022). Land Statistics and Indicators. Global, Regional and Country Trends, 2000–2020. Faostat Analytical Brief 48. https://www.fao.org/3/cc0963en/cc0963e.

[3] FAO (2006). Livestock’s Long Shadow. Environmental Issues and Options.

[4] Risner D., Kim Y., Nguyen C., Siegel J.B., Spang E.S. (2023). Environmental impacts of cultured meat: A cradle-to-gate life cycle assessment. bioRxiv.

[5] Shapiro P. (2018). Clean meat: How growing meat without animals will revolutionize dinner and the world. Science.

[6] Bhat Z.F., Fayaz H. (2011). Prospectus of cultured meat—Advancing meat alternatives. J. Food Sci. Technol..

[7] Kumar P., Sharma N., Sharma S., Mehta N., Verma A.K., Chemmalar S., Sazili A.Q. (2021). In-vitro meat: A promising solution for sustainability of meat sector. J. Anim. Sci. Technol..

[8] Mauro A. (1961). Satellite cell of skeletal muscle fibers. J. Biophys. Biochem. Cytol..

[9] Chargé S.B.P., Rudnicki M.A. (2004). Cellular and Molecular Regulation of Muscle Regeneration. Physiol. Rev..

[10] Chal J., Pourquié O. (2017). Making muscle: Skeletal myogenesis in vivo and in vitro. Development.

[11] Kuang S., Rudnicki M.A. (2008). The emerging biology of satellite cells and their therapeutic potential. Trends Mol. Med..

[12] Will K., Schering L., Albrecht E., Kalbe C., Maak S. (2015). Differentiation of bovine satellite cell-derived myoblasts under different culture conditions. Vitro Cell. Dev. Biol.-Anim..

[13] Dodson M.V., Martin E.L., Brannon M.A., Mathison B.A., McFarland D.C. (1987). Optimization of bovine satellite cell-derived myotube formation in vitro. Tissue Cell..

[14] Kowalski K., dos Santos M., Maire P., Ciemerych M.A., Brzoska E. (2018). Induction of bone marrow-derived cells myogenic identity by their interactions with the satellite cell niche. Stem Cell Res. Ther..

[15] Musarò A., Barberi L. (2010). Isolation and culture of mouse satellite cells. Methods Mol. Biol..

[16] Zhang J., Li Q., Yan Y., Sun B., Wang Y., Tang L., Wang E., Yu J., Nogoy K.M.C., Li X. (2021). Effect of ciglitazone on adipogenic transdifferentiation of bovine skeletal muscle satellite cells. J. Anim. Sci. Technol..

[17] Guthridge M., Wilson M., Cowling J., Bertolini J., Hearn M.T.W. (1992). The Role of Basic Fibroblast Growth Factor in Skeletal Muscle Regeneration. Growth Factors.

[18] Yasui R., Sekine K., Taniguchi H. (2021). Clever Experimental Designs: Shortcuts for Better iPSC Differentiation. Cells.

[19] Kolkmann A.M., Van Essen A., Post M.J., Moutsatsou P. (2022). Development of a Chemically Defined Medium for in vitro Expansion of Primary Bovine Satellite Cells. Front. Bioeng. Biotechnol..

[20] Li J., Gonzalez J.M., Walker D.K., Hersom M.J., Ealy A.D., Johnson S.E. (2011). Evidence of heterogeneity within bovine satellite cells isolated from young and adult animals. J. Anim. Sci..

[21] Lyu P., Settlage R.E., Jiang H. (2021). Genome-wide identification of enhancers and transcription factors regulating the myogenic differentiation of bovine satellite cells. BMC Genom..

[22] Dai Y., Wang Y.M., Zhang W.R., Liu X.F., Li X., Ding X.B., Guo H. (2016). The role of microRNA-1 and microRNA-206 in the proliferation and differentiation of bovine skeletal muscle satellite cells. Vitro Cell. Dev. Biol.-Anim..

[23] Shahini A., Vydiam K., Choudhury D., Rajabian N., Nguyen T., Lei P., Andreadis S.T. (2018). Efficient and high yield isolation of myoblasts from skeletal muscle. Stem Cell Res..

[24] Pezzanite L., Chow L., Griffenhagen G., Dow S., Goodrich L. (2021). Impact of Three Different Serum Sources on Functional Properties of Equine Mesenchymal Stromal Cells. Front. Vet. Sci..

[25] Franke J., Abs V., Zizzadoro C., Abraham G. (2014). Comparative study of the effects of fetal bovine serum versus horse serum on growth and differentiation of primary equine bronchial fibroblasts. BMC Vet. Res..

[26] Schindelin J., Arganda-Carreras I., Frise E., Kaynig V., Longair M., Pietzsch T., Preibisch S., Rueden C., Saalfeld S., Schmid B. (2012). Fiji: An open-source platform for biological-image analysis. Nat. Methods.

[27] CLAHE (Contrast Limited Adaptive Histogram Equalization). https://imagej.nih.gov/ij/plugins/clahe/index.html.

[28] Van Rossum G., Drake F.L. (2009). Python 3 Reference Manual.

[29] Harris C.R., Millman K.J., van der Walt S.J., Gommers R., Virtanen P., Cournapeau D., Wieser E., Taylor J., Berg S., Oliphant T.E. (2020). Array programming with NumPy. Nature.

[30] Virtanen P., Gommers R., Oliphant T.E., Haberland M., Reddy T., Cournapeau D., Burovski E., Peterson P., Weckesser W., Bright J. (2020). SciPy 1.0. Fundamental Algorithms for Scientific Computing in Python. Nat. Methods.

[31] Seabold S., Perktold J. Statsmodels: Econometric and statistical modeling with python. Proceedings of the 9th Python in Science Conference.

[32] McKinney W. Data structures for statistical computing in python. Proceedings of the 9th Python in Science Conference.

[33] The Pandas Development Team (2020). Pandas-Dev/Pandas: Pandas. Zenodo. https://zenodo.org/record/7979740.

[34] Hunter J.D. (2007). Matplotlib: A 2D Graphics Environment. Comput. Sci. Eng..

[35] Woods T.L., Smith C.W., Zeece M.G., Jones S.J. (1997). Conditions for the culture of bovine embryonic myogenic cells. Tissue Cell.

[36] Soleimani V.D., Punch V.G., Kawabe Y., Jones A.E., Palidwor G.A., Porter C.J., Cross J.W., Carvajal J.J., Kockx C.E.M., van IJcken W.F.J. (2012). Transcriptional Dominance of Pax7 in Adult Myogenesis Is Due to High-Affinity Recognition of Homeodomain Motifs. Dev. Cell..

[37] Hinterberger T.J., Sassoon D.A., Rhodes S.J., Konieczny S.F. (1991). Expression of the muscle regulatory factor MRF4 during somite and skeletal myofiber development. Dev. Biol..

[38] Lazure F., Blackburn D.M., Corchado A.H., Sahinyan K., Karam N., Sharanek A., Nguyen D., Lepper C., Najafabadi H.S., Perkins T.J. (2020). Myf6/MRF4 is a myogenic niche regulator required for the maintenance of the muscle stem cell pool. EMBO Rep..

[39] Pawlikowski B., Vogler T.O., Gadek K., Olwin B.B. (2017). Regulation of skeletal muscle stem cells by fibroblast growth factors. Dev. Dyn..

[40] Park J., Lee J., Song K.D., Kim S.J., Kim D.C., Lee S.C., Son Y.J., Choi H.W., Shim K. (2021). Growth factors improve the proliferation of Jeju black pig muscle cells by regulating myogenic differentiation 1 and growth-related genes. Anim. Biosci..

[41] Furuichi Y., Kawabata Y., Aoki M., Mita Y., Fujii N.L., Manabe Y. (2021). Excess Glucose Impedes the Proliferation of Skeletal Muscle Satellite Cells Under Adherent Culture Conditions. Front. Cell Dev. Biol..

[42] Aguiari P., Leo S., Zavan B., Vindigni V., Rimessi A., Bianchi K., Franzin C., Cortivo R., Rossato M., Vettor R. (2008). High glucose induces adipogenic differentiation of muscle-derived stem cells. Proc. Nat. Acad. Sci. USA.

[43] Kolkmann A.M., Post M.J., Rutjens M.A.M., van Essen A.L.M., Moutsatsou P. (2020). Serum-free media for the growth of primary bovine myoblasts. Cytotechnology.

[44] Stout A.J., Mirliani A.B., Rittenberg M.L., Shub M., White E.C., Yuen J.S.K., Kaplan D.L. (2022). Simple and effective serum-free medium for sustained expansion of bovine satellite cells for cell cultured meat. Commun. Biol..

